# Correlates of felt age in caregivers of people with dementia: findings from the IDEAL study

**DOI:** 10.3389/fpsyg.2023.1287842

**Published:** 2024-01-12

**Authors:** Serena Sabatini, Shelbie G. Turner, Robin G. Morris, Carol Opdebeeck, Jeanette M. Thom, Anna Hunt, Louise Allan, Claire Pentecost, Linda Clare

**Affiliations:** ^1^Institute of Mental Health, School of Medicine, University of Nottingham, Nottingham, United Kingdom; ^2^Division of Geriatrics and Palliative Medicine, Weill Cornell Medical College, New York, NY, United States; ^3^Department of Psychology, King's College London Institute of Psychiatry, Psychology and Neuroscience, London, United Kingdom; ^4^Department of Psychology, Manchester Metropolitan University, Manchester, United Kingdom; ^5^School of Health Sciences, The University of Sydney, Darlington, NSW, Australia; ^6^REACH: The Centre for Research in Ageing and Cognitive Health, University of Exeter Medical School, University of Exeter, Exeter, United Kingdom; ^7^NIHR Applied Research Collaboration South-West Peninsula, Exeter, United Kingdom

**Keywords:** subjective age, self-perceptions of aging, views on aging, dementia caregiving, carers

## Abstract

**Objective:**

Family relationships influence how people appraise their own aging and how their appraisals impact their health. We analyzed felt age (FA) among family caregivers of people with dementia.

**Methods and measures:**

We used a stratified sample of 1,020 spousal and 202 adult-child caregivers from the IDEAL study. We estimated cross-sectional associations and bidirectional influences between caregivers' FA and their health and wellbeing (depression, number of health conditions, stress, positive aspects of caregiving) over 2 years.

**Results:**

Among spousal caregivers, 25% had a younger FA and 36% had an older FA. Among adult-child caregivers, 21.8% had a younger FA and 36.1% had an older FA. In spousal and adult-child caregivers an older FA was cross-sectionally associated with higher depression, number of health conditions, and stress, and fewer positive aspects of caregiving. In spousal caregivers, hours of care per day moderated the association between FA and depression, and FA was associated with stress 1 year later.

**Conclusion:**

Caregiving may impact FA and its relationship with health. We urge continued research on the connections between caregiving and FA, and how interventions might support caregivers' positive views on their own aging, which will translate views on aging scholarship to meaningfully improve caregivers' lives.

## 1 Introduction

The literature on people's subjective appraisals of their own aging processes—their Views on Aging (VoA)—and the influence VoA have on health and wellbeing has grown immensely in the past two decades. New constructs have emerged to capture the multi-faceted and domain-specific nature of VoA, such as *Awareness of Age-Related Change* (Diehl et al., 2014) and *Future Self-Views* (Kornadt et al., 2020). However, the question of how old people feel relative to their chronological age, often empirically measured via a single item, continues to be a reliable and precise predictor of healthy aging processes. Thus, felt age (FA), as we refer to it throughout this paper, remains the focus of much psychosocial and biological aging research.

One way of assessing FA is to ask participants to report whether they feel younger than, the same as, or older than their chronological age (Barak and Stern, 1986; Montepare, 2020). Research suggests that the majority of middle-aged and older adults feel younger than their chronological age (i.e., younger FA) (Montepare and Lachman, 1989; Westerhof et al., 2003, 2014; Rubin and Berntsen, 2006; Choi and DiNitto, 2014; Kotter-Grühn et al., 2016; Bordone et al., 2019; Opdebeeck et al., 2019). This finding has held true over time and is consistent across different sample populations. For example, a 2003 study suggested that both Germans and Americans tended to have younger FAs (Westerhof et al., 2003). In 2014, researchers analyzing the United States' National Health and Aging Trends Study found that over 70% of people aged 65–79 years old had younger FAs (Choi and DiNitto, 2014). And, in a study of cognitively healthy older adults in the United Kingdom (UK), 77.5% reported a younger FA; 12.4% reported feeling as old as their age (age-congruent FA), and 10.2% reported feeling older than their age (older FA) (Sabatini et al., 2021a). Moreover, a recent meta-analysis comprising 294 studies from 148 countries found that with increasing age, people feel increasingly younger than their chronological age (Pinquart and Wahl, 2021).

The majority of middle-aged and older adults who have younger FAs benefit from feeling younger than they actually are. Middle-aged and older adults who feel younger than their chronological age engage in more health promotive behaviors such as physical activity, getting enough sleep and high quality sleep, and even flossing their teeth (Caudroit et al., 2012; Wienert et al., 2015; Montepare, 2020; Sabatini et al., 2021b). Likely a result of better health behavior, younger FA is associated with healthier physiology such as better liver and kidney functioning (Thyagarajan et al., 2019).

However, just as a younger FA is a protective factor for health maintenance, an older FA is a risk factor for poorer health. Indeed, the smaller proportions of middle-aged and older adults having an age-congruent or older FA generally experience poorer physical (Stephan et al., 2013; Veenstra et al., 2020; Sabatini et al., 2021a), mental (Montepare and Lachman, 1989; Sabatini et al., 2020), and cognitive (Stephan et al., 2014; Kwak et al., 2018; Debreczeni and Bailey, 2021) health. Recent studies using United States nationally-representative samples found that, among adults aged 50 years and older, those who had older FAs had more physiological markers of inflammation (Stephan et al., 2022), and a greater likelihood of heart disease and stroke (Skoblow and Proulx, 2022). An older FA is also associated with greater risks of hospitalization (Stephan et al., 2016) and mortality (Uotinen et al., 2005; Stephan et al., 2018).

### 1.1 Felt age and family relationships

Given the saliency of VoA to health behaviors and outcomes, researchers are working to identify factors that contribute to people's appraisals of their own aging, including what makes them feel older than, younger than, or the same as their actual age. Researchers have identified family relationships, especially the relationship between spouses and partners, as key drivers of the VoA construct *Self-Perceptions of Aging* (Mejía and Gonzalez, 2017; Kim et al., 2018; Mejía et al., 2020; Rupprecht and Lang, 2020; Turner et al., 2022). To date, however, there is not as much scholarship on how family relationships influence FA, nor how family relationships contribute to FA's connection to health. Continuing to explore how family relationships are connected to FA, especially as one or both relationship partners navigate age-related or health changes, is crucial to further understand both FA specifically and VoA more broadly, including how VoA and their associations with health change over time.

### 1.2 Felt age in dementia family caregivers

In this study, we focused on caring for a spouse or parent with dementia as a family relationship dynamic that could impact both caregivers' FA and FA's association with caregivers' health and wellbeing. Researchers are just starting to explore dementia family caregivers' VoA and their impact on caregivers' health outcomes (Turner et al., 2023). To our knowledge there are no studies that specifically focus on dementia family caregivers' FA. The frequency of older and younger FAs among caregivers of people with dementia is unknown, as is the extent to which caregiving impacts the associations between FA and health and wellbeing over time.

Diehl et al. (2014) heuristic model of Awareness of Aging in the Context of Life-Span Developmental Processes and Outcomes includes developmental influences, such as life events and experiences, as contributors to the development of FA, and as influencers of the ways in which FA impacts health behavior and health outcomes. For many, caring for an aging relative is a major life event. Some scholars now even consider it a “defining feature” of mid-life and older adulthood (Infurna et al., 2020). Thus, per Diehl et al.' (2014) model, the life event of caregiving may influence a caregiver's FA. Specifically, the nature of the caregiving situation—such as the intensity of care—may shape caregivers' FA and may moderate the relationship between caregivers' FA and health.

Notably, the Diehl et al. (2014) model, alongside recent empirical scholarship on VoA (Wettstein et al., 2021; Sabatini et al., 2023a), suggests that various VoA constructs are reciprocally connected to health. That is, just as VoA are predictors of longer-term health, health is also a predictor of longer-term VoA. As such, just as caregivers' FA might impact their health, their health may impact their FA. Recent evidence suggests that these bidirectional associations between VoA and aspects of physical and mental health may be consistently present across age groups (Sabatini et al., 2023a). Caregivers can have worse mental and physical health than non-caregivers, including more instances of severe stress, depression, and anxiety, as well as physical morbidities such as pain, hypertension, and diabetes (Janson et al., 2022). Moreover, there is great variability in physical and mental health between different types of caregivers and caregiving situations. Adult-child caregivers tend to report more depressive symptoms than spousal caregivers, for example (Watson et al., 2019). Caregivers who care for more hours per day (Wulff et al., 2020), and who report greater negative subjective appraisals of caregiving (Macchi et al., 2020; Nah et al., 2022) have poorer mental health, which is a contributor to feeling older than one's chronological age (Kotter-Grühn et al., 2015). Likewise, caregivers greater negative subjective appraisals of caregiving have worse physical health (Talley and Crews, 2007; Liang et al., 2020; Snyder and Vitaliano, 2020), which is associated with having an older FA (e.g., Veenstra et al., 2020). Taken together, this evidence suggests that the physical and psychological stressors caregivers face may impact their FA over time. Analyzing this possibility is important not only to more thoroughly understand which caregivers may be especially at risk of feeling older than their chronological age but also to offer insight on a potential psychological pathway linking caregiving to longer term health outcomes.

### 1.3 The proposed study

We used baseline, 12-month, and 24-month follow-up data from a large sample of spousal (including partners) and adult-child caregivers of community-dwelling people with mild-to-moderate dementia at baseline to address four aims. First, we documented FA in spousal and adult-child caregivers and how FA changed over 2 years. Second, we analyzed how FA was cross-sectionally associated with various health and wellbeing indicators. Third, we analyzed how the number of hours of caregiving per day moderated the associations of FA with each health and wellbeing indicator. Fourth, we explored the effects that FA and each health and wellbeing indicator had on each other between one timepoint and the next (i.e., bidirectional influences).

We had the following hypotheses. First, we expected that caregivers' FA would become more negative over time due to the increasing caregiving demands associated with the progress of dementia in care receivers. Second, consistently with research in non-caregivers, we expected that an older FA would be negatively associated with health and wellbeing indicators (Westerhof et al., 2023). Third, we expected that an older FA would have a greater negative effect on health and wellbeing indicators among those caregivers providing more hours of care per day, under the premise that those caregivers who provide more hours of care are at greater risk of poor health and wellbeing (Wulff et al., 2020). Fourth, consistent with previous evidence on VoA in the general population (Wettstein et al., 2021; Sabatini et al., 2023a) and with theoretical models of VoA (Diehl et al., 2014), we expected bidirectional influences between FA and health and wellbeing indicators.

## 2 Materials and methods

This study used data for caregivers of people with dementia collected in the first three waves (baseline: 2014–16; 12-month follow-up: 2015–17; and 24-month follow-up: 2016–18) of the Improving the experience of Dementia and Enhancing Active Life (IDEAL) study (Clare et al., 2014). We used version 7 of the datasets. IDEAL was approved by the Wales 5 Research Ethics Committee (reference: 13/WA/0405) and the Ethics Committee of the School of Psychology, Bangor University (reference: 2014-11684). IDEAL is registered with UK Clinical Research Network, registration No. 16593.

In IDEAL, people with dementia were recruited through a network of 29 National Health Service (NHS) sites in England, Scotland, and Wales. At baseline, participants had to live at home, have a confirmed diagnosis of dementia of any subtype, and have mild-to-moderate dementia as indicated by a Mini-Mental State Examination (Folstein et al., 1975) score ≥15. We refrained from using a MMSE upper limit because some people can have a perfect MMSE score and a diagnosis of dementia as MMSE is influenced by education level (Shiroky et al., 2007). In IDEAL, at baseline 30% of people with dementia has a MMSE score ≥25. In this study sample at baseline 55% caregivers were caring for a person with dementia who received a diagnosis within less than a year and 33% caregivers were caring for a person with dementia who received a diagnosis between one and 2 years ago. In these initial stages of dementia, high scores on the MMSE are to be expected. Moreover, all people with dementia who enrolled in the IDEAL study received a clinical diagnosis of dementia which was confirmed at later timepoints. In addition, as expected in people with a progressive disease like dementia, cognitive functioning declined in IDEAL participants during the 2-year study period.

Exclusion criteria were having a co-morbid terminal illness at baseline, inability to provide informed consent at baseline, and/or any known potential for home visits to pose risk to research staff.

When a person with dementia joined the IDEAL study, if they had a caregiver and agreed for the caregiver to be invited, investigators invited the caregiver to participate in the IDEAL study as well. Caregivers were defined as family members or friends who provided unpaid caregiving (i.e., practical or emotional support) for the person with dementia (Quinn et al., 2020). There were no specific inclusion criteria for caregivers, other than being willing and available to take part. At baseline the IDEAL cohort comprised 1,277 caregivers. We excluded caregivers who did not answer the one-item FA question at any of the three timepoints (265 caregivers), or ceased their caring role over the study period (54 additional caregivers). We also excluded caregivers who were not either spouses/partners or adult children/stepchildren/children-in-law (from now on referred to as adult children or adult-child caregivers) of the person with dementia (23 additional caregivers). The resulting baseline sample for analyses included 1,203 caregivers of people with mild-to-moderate dementia; 1,006 were spouses or partners of the person with dementia (hereafter called spousal caregivers), and 197 were adult children of the person with dementia (hereafter called adult-child caregivers). The 12-month follow-up sample comprised 803 spousal caregivers and 136 adult-child caregivers, and the 24-month follow-up sample comprised 605 spousal caregivers and 96 adult-child caregivers.

### 2.1 Participants

At baseline, spousal caregivers were, on average, 72.4 years old (*SD* = 8.2); 66.5% were women. Almost all were White British (96.3%). A minority (3%) were of other White ethnicities and 0.7% were of other non-White ethnicities including Caribbean, African, Indian, Pakistani, and other mixed backgrounds. At baseline, adult-child caregivers were, on average, 52.98 years old (*SD* = 8.4 years), and 80.2% were women. Almost all were White British (96.5%), 1% were of other White ethnicities, and 2.5% were of other non-White ethnicities including Caribbean, African, Indian, Pakistani, and other mixed backgrounds.

Results from analysis of variance (ANOVA) (Table 1) suggest that at baseline, compared to adult-child caregivers, spousal caregivers were older, included a lower proportion of women, were less educated, and included a higher proportion of white participants. These differences remained significant at follow-ups, except for ethnicity which was no longer significantly different at follow-ups.

**Table 1:** Sociodemographic characteristics of participants at baseline, 12-months, and 24-months follow-ups.

	**Spousal Caregivers**			**Adult-child caregivers**			**Comparison of spousal and adult-child caregivers**					
**Caregivers' variables (except age of care recipient)**	**Baseline (*****N*** = **1,006)**	**12-month follow-up (N** = **803)**	**24-month follow-up (N** = **605)**	**Baseline (*****N*** = **197)**	**12-month follow-up (N**= **136)**	**24-month follow-up (N**= **96)**	**Baseline** ***p*****-value**	**Eta-squared**	**12-month follow-up**	**Eta-squared**	**24-month follow-up**	**Eta-squared**
Age in years, Mean (SD; range)	72.37 (8.22; 41–92)	73.19 (7.93; 42–93)	73.76 (7.85; 43–94)	52.98 (8.42; 26–89)	53.67 (8.05; 27–71)	54.25 (8.0; 31–68)	< 0.001	0.4300	< 0.001	0.4300	< 0.001	0.4200
Age of care recipient, Mean (SD; range)	75.00 (7.80; 43–95)	75.77 (7.62; 50–92)	76.24 (7.74; 51–93)	81.55 (7.47; 53.98)	82.76 (7.08; 60–96)	83.07 (7.28; 61–97)	< 0.001	0.0900	< 0.001	0.1000	< 0.001	0.0900
Age difference between caregiver and care recipient, Mean (SD)	2.62 (5.34)	2.57 (5.31)	2.48 (5.18)	28.57 (7.34)	29.10 (5.86)	28.82 (5.82)	< 0.001	0.7400	< 0.001	0.7500	< 0.001	0.7500
Sex: Women, *n* (%)	669 (66.5)	533 (66.4)	396 (65.5)	158 (80.2)	113 (83.1)	80 (83.3)	< 0.001		< 0.001		< 0.001	
Education, Mean (SD)	2.51 (1.10)	2.54 (1.10)	2.57 (1.10)	3.09 (0.88)	3.16 (0.89)	3.18 (0.89)	< 0.001	0.0400	< 0.001	0.0400	< 0.001	0.0400
Missing, *n*	3	2	2	3	2	1						
Ethnicity, *n* (%)												
White British	969 (96.3)	775 (96.5)	583 (96.4)	190 (96.5)	133 (97.8)	94 (98.0)	0.018		0.267		0.514	
White other	30 (3.0)	22 (2.7)	18 (3.0)	2 (1.0)	1 (0.7)	1 (1.0)						
Other	7 (0.7)	6 (0.8)	4 (0.6)	5 (2.5)	2 (1.5)	1 (1.0)						
Hours of caregiving per day^*^, Mean (SD)	1.23 (0.77)	1.33 (0.73)	1.38 (0.70)	0.92 (0.65)	0.98 (0.66)	0.91 (0.67)	< 0.001	0.0200	< 0.001	0.0300	< 0.001	0.0500
Missing, *n*	10	21	5	1	0	2						
Felt age, Mean (SD)	4.36 (1.72)	4.41 (1.71)	4.39 (1.71)	4.45 (1.77)	4.14 (1.80)	4.32 (1.66)	0.511	0.0003	0.105	0.0030	0.713	0.0002
Missing, *n*	6	42	26	0	9	1						
Depression, Mean (SD)	7 (7.55)	8.32 (9.0)	8.80 (9.16)	8.02 (9.62)	9.73 (10.92)	9.25 (11.95)	0.107	0.0020	0.118	0.0030	0.674	0.0003
Missing	50	62	45	7	13	5						
Number of health conditions, Mean (SD)	1.46 (1.43)	1.85 (1.66)	2.07 (1.85)	0.82 (1.26)	1.05 (1.44)	0.99 (1.34)	< 0.001	0.0300	< 0.001	0.0300	< 0.001	0.0400
Missing, n	56	36	36	10	6	2						
Stress, mean (SD)	19.33 (9.77)	22.17 (10.10)	23.29 (10.13)	18.88 (10.13)	20.08 (9.13)	21.75 (10.15)	0.574	0.0003	0.031	0.0100	0.176	0.0030
Missing, *n*	48	54	42	10	11	3						
Positive aspects of caregiving, Mean (SD)	28.01 (7.45)	27.72 (7.75)	27.73 (7.82)	29.56 (6.90)	30.05 (7.42)	29.41 (6.88)	0.007	0.0100	0.002	0.0100	0.052	0.0100
Missing, *n*	24	50	33	2	9	3						

*p*-values calculated using chi-squared tests for categorical variables and analysis of variance (ANOVA) for continuous variables. For eta-squared (η2) effect sizes between 0.01 and 0.05 are interpreted as small, between 0.06 and 0.13 are interpreted as moderate and of 0.14 or above are interpreted as large (Cohen, 1988). Eta-squared are reported solely for continuous and ordinal variables. SD, Standard deviation. N/n, Number. ^*^For analyses purposes hours of caregiving per day was treated as an ordinal variable and in the current table descriptive statistics are reported for the hours of caregiving as an ordinal variable.

#### 2.1.1 Attrition

Supplementary Table 1 reports descriptive statistics for study variables at baseline for those who participated at 1-year follow-up and those who left the study between baseline and 1-year follow-up. ANOVAs suggest that these two groups differed in some study variables. Specifically, compared to those who provided data at one-year follow-up, those who left the study between baseline and 1-year follow-up were more likely to provide care for more hours per day, to have fewer health conditions, and to report higher levels of stress.

Supplementary Table 2 reports descriptive statistics of study variables at 1-year follow-up for those who participated at 2-year follow-up and those who left the study between 1-year follow-up and 2-year follow-up. ANOVAs suggest that these two groups differed in some study variables. Specifically, compared to those who provided data at 2-year follow-up, those who left the study between 1-year and 2-year follow-up were more likely to provide care for more hours per day and to report higher levels of stress.

### 2.2 Measures

For the purpose of current study analyses, the following measures were selected from the wider IDEAL dataset. All measures (paper and pencil format) were self-completed in person and administered at all timepoints by trained researchers (i.e., annual training was provided before data collection began). The assessment procedure was standardized among administering centers and measures were administered in the same order to all participants. Measure administration was conducted over in three in-person visits at baseline and in two in-person visits at follow-up.

#### 2.2.1 Felt age

Caregivers reported their FA by answering the single-item question “*How old do you feel at the moment?”* (Opdebeeck et al., 2019; Montepare, 2020). Participants selected one of the following response items: 1 = “a lot older than my age,” 2 = “a little older,” 3 = “not much older,” 4 = “about the same,” 5 = “not much younger,” 6 = “a little younger,” 7 = “a lot younger than my age.” This variable was treated as ordinal in the study analyses. This measure was administered during the second visit both at baseline and follow-ups.

#### 2.2.2 Depression

Caregivers responded to the 20-item screening tool Center for Epidemiologic Studies Depression Scale-Revised Short Form (Eaton et al., 2004) as a measure of depression. A sample item is “*I was bothered by things that usually don't bother me*.” For each of the 20 items participants indicate the frequency of its occurrence (0 = “not at all or < 1 day last week,” 1 = “1–2 days last week,” 2 = “3–4 days last week,” 3 = “5–7 days last week,” or “nearly every day for 2 weeks”). Item scores are summed up to give a total score. Scores could range from 0–60; higher scores indicate greater depression. Cronbach's alpha was 0.89 at baseline, 0.91 at 12-month follow-up, and 0.82 at 24-month follow-up indicating excellent internal consistency. The original validation study of the Relative Stress Scale obtained a Cronbach's alpha of 0.92, indicating excellent internal consistency (Eaton et al., 2004). This measure was administered during the second visit both at baseline and follow-ups.

#### 2.2.3 Number of health conditions

We measured caregivers' health conditions via the Charlson Comorbidity Index (Charlson et al., 1987, 2008). Number of health conditions was a count of heart problems (heart attack or congestive heart failure), hypertension, peripheral vascular disease, stroke, hemiplegia, transient ischaemic attack, chronic bad chest, inflammation of the joints, peptic/stomach ulcer disease, skin ulcer, diabetes, moderate or severe kidney disease, cancer, and liver disease. All conditions were assigned a score of one when present and a score of zero when not present. The total score is the sum of the health conditions the participant reported; higher scores indicate a higher number of health conditions.

The CCI is reliable and valid for diverse clinical cohorts (e.g., people with cancer, amputation, and arthritis) in a variety of healthcare settings (de Groot et al., 2003; Hall et al., 2004; Quan et al., 2011; Roffman et al., 2014). This measure was administered during the third visit at baseline and during the second visit at follow-ups.

#### 2.2.4 Stress

We measured caregivers' stress via the 15-item Relative Stress Scale (Greene et al., 1993). A sample question is “*Do you ever feel that you need a break?”* Participants responded on a Likert scale ranging from 1 = “never” to 5 = “always.” Scores could range from 0 to 60, with higher scores indicating greater stress. Cronbach's alpha was 0.89 at baseline, 0.89 at 12-month follow-up, and 0.90 at 24-month follow-up indicating excellent internal consistency. The original validation study of the Relative Stress Scale obtained a Cronbach's alpha of 0.85, indicating good internal consistency (Greene et al., 1982). This measure was administered during the second visit both at baseline and follow-ups.

#### 2.2.5 Positive aspects of caregiving

Caregivers reported how positively they felt about caregiving via the nine-item Positive Aspects of Caregiving scale (Tarlow et al., 2004). A sample question includes: “*Providing help to my relative/friend has made me feel more useful.”* Participants responded on a Likert scale ranging from 1 = “disagree a lot” to 5 “agree a lot.” Scores could range from 9 to 45, with higher scores indicating more positive experiences of caring. Cronbach's alpha was 0.91 at baseline, 0.92 at 12-month follow-up, 0.92 at 24-month follow-up indicating excellent internal consistency. The original validation study of the Positive Aspects of Caregiving scale obtained a Cronbach's alpha of 0.89, hence also indicating excellent internal consistency (Tarlow et al., 2004). This measure was administered during the second visit both at baseline and follow-ups.

#### 2.2.6 Hours of care per day

Hours of caregiving per day was assessed with a categorical variable: < 1 hour of caregiving per day; 1 to 10 h of caregiving per day; more than 10 h of caregiving per day. This variable was treated as ordinal in the study analyses. This variable was used in the study as an indicator of dementia severity. This measure was administered during the first visit at baseline and follow-ups.

#### 2.2.7 Personal characteristics

Caregiver demographic characteristics included age, age groups (< 65; 65–69; 70–74; 75–79; 80+ years), sex, and education level. Education level was classified into four groups: no qualifications, school leaving certificate at age 16, school leaving certificate at age 18, and university level education. We treated education as an ordinal variable in study analyses. Additional demographic characteristics include the age of the care recipient and the age difference between the caregiver and the care recipient (though we included these variables only in correlational analyses). These measures were administered during the first visit at baseline and follow-ups.

### 2.3 Analyses

Due to differences in the number of participants, socio-demographic characteristics, and study variables between spousal and adult-child caregivers, we conducted parallel analyses for spousal and adult-child caregivers. In this sample, only 2 caregivers were step-children and only 6 were son-daughter in law, so we did not have the power to investigate differences between adult-child caregivers and step-children and son-daughters in law. For both subsamples, we provided descriptive statistics of study variables at all timepoints, including both the mean of FA and the frequencies of various FA categories among caregivers. We also provided results of chi-squared tests (for categorical variables) and ANOVA (for continuous variables) investigating differences in these two subsamples. Moreover, we provided descriptive statistics for study variables at baseline for those who stayed in the study until 1-year follow-up and those who dropped out between baseline and 1-year follow-up. Similarly, we provided descriptive statistics for study variables at 1-year follow-up for those who stayed in the study until 2-year follow-up and for those who dropped out between 1-year follow-up and 2-year follow-up. We used chi-squared tests (for categorical variables) and ANOVA (for continuous variables) to test differences in these two subsamples.

We estimated Pearson's *r* correlation coefficients between key study variables (i.e., age, FA, care recipient age, age difference between caregiver and care recipient, sex, education, hours of caregiving per day, number of health conditions, depression, stress, and positive aspects of caregiving) in the overall study sample at baseline, as well as in the two subsamples of spousal and adult-child caregivers at baseline, 12-month follow-up, and 24-month follow-up. Correlation coefficients ≤ 0.09 were considered negligible, between 0.10 and 0.29 were considered small, between 0.30 and 0.49 were considered moderate, and ≥0.50 were considered large (Cohen, 1988). Statistical significance after having applied Bonferroni's correction was set at *p* < 0.001.

We used multilevel models to investigate change over time in FA and health and wellbeing variables. We first estimated Intraclass Correlation Coefficients (ICC) for FA, depression, health conditions, stress, and positive aspects of caregiving. For FA, depression, stress, and positive aspects of caregiving, we reported mean intercept (baseline) and mean slope (change over time) with 95% confidence intervals (CI). For number of health conditions, we specified a Poisson distribution, and we reported Incidence Rate Ratios (IRR) and 95% CI. We then tested whether the associations between FA and health and wellbeing variables were moderated by hours of caregiving per day at baseline. In the analyses age, sex, and education were treated as covariates as they can have an effect on FA (Henderson et al., 1995; Pinquart and Wahl, 2021; Sabatini et al., 2021a) and they may have an effect on study outcomes (e.g., number of health conditions) (Covinsky et al., 2003; World Health Organization, 2021, 2023).

To explore bidirectional associations (i.e., whether two variables have an effect on each other from one timepoint to the next) of FA with each of depression, number of health conditions, stress, and positive aspects of caregiving, we fit Random Intercepts-Cross Lagged Panel Models (RI-CLPMs) (Hamaker et al., 2015; Orth et al., 2021). RI-CLPMs account for trait-like, time-invariant stability through the inclusion of a random intercept (i.e., a factor with all loadings constrained to 1). This random intercept decomposes within-person (WP) and between-person (BP) variance; making it possible to overcome the limitations of traditional CLPMs which account only for temporal stability through the inclusion of autoregressive parameters (Hamaker et al., 2015, see Burns et al., 2020 for comparison of CLPM with RI-CLPM). As indicators of model fit, we reported the Comparative Fit Index (CFI), the Root Mean Square Error of Approximation (RMSEA), the Standardized Root Mean Square Residual (SRMR), and the Tucker-Lewis index (TLI). Criteria for acceptable model fit were CFI and TLI >0.90, RMSEA < 0.08 (90% CI: between 0 and 0.08), and SRMR < 0.06 (Byrne, 2012). Our predictor, felt age, was normally distributed. Scores on the relative stress scale and positive aspects of caregiving (outcomes in this study) were normally distributed. Depression and number of health conditions were not normally distributed. However, estimates remained similar when specifying a positively skewed distribution. We conducted the RI-CLPMs analyses in Mplus (Muthén and Muthén, 2017) and all other analyses in STATA version 17 (StataCorp, 2021).

## 3 Results

### 3.1 Descriptive statistics

Descriptive statistics for all study variables for spousal and adult-child caregivers are available in Table 1. Results from ANOVA comparing spousal caregivers and adult-child caregivers (also reported in Table 1) suggest that at baseline, spousal caregivers provided care for more hours per day, had more health conditions, and reported fewer positive aspects of caregiving. These differences remained significant at both 12- and 24-month follow-ups, except for stress, which was significantly higher, and positive aspects of caregiving, which were significantly lower, among spousal caregivers at the 12-month follow-up but not at the 24-month follow-up.

Although in the study analyses we treated FA as an ordinal variable, here we report descriptive statistics for FA categories in order to better understand differences in the FA of the two subsamples, as well as to enable comparability of our results with other studies that use categorical measures of FA. At baseline 39% of spousal caregivers felt about the same as their age; 2.7, 1.2, and 14.1% felt not much younger than their age, a little younger than their age, and a lot younger than their age, respectively. Additionally, 0.1, 12.4, and 7.5% felt a lot older than their age, a little older than their age, and not much older than their age, respectively. At baseline 35.5% of adult-child caregivers felt about the same as their age; 5.1, 25.4, and 12.1% felt not much younger than their age, a little younger than their age, and a lot younger than their age, respectively. Additionally, 7.1, 11.7, and 3.1% felt a lot older than their age, a little older than their age, and not much older than their age, respectively. An ANOVA testing differences in mean levels of FA between spousal and adult-child caregivers showed that spousal caregivers, on average, felt older than their chronological age to a greater extent than did adult-child caregivers.

At baseline in the overall sample, a younger FA was correlated with older chronological age of the caregiver, older chronological age of the care recipient, smaller difference between the age of the care recipient and the caregiver, being male, greater education, fewer hours of caregiving per day, fewer health conditions, lower depression, lower stress, and more positive aspects of caregiving (Table 2). However, some correlations (i.e., chronological age of the caregiver, hours of caregiving per day, depression, and stress), though significant, were very small. Correlations among key study variables at baseline, 12-month follow-up, and 24-month follow-up stratified for spousal and adult-child caregivers can be found in Supplementary Tables 3–5, respectively.

**Table 2:** Correlation matrix of key study variables at baseline for the overall study sample.

	**Mean**	**SD**	**Pearson's** ***r*** **correlation coefficient**									
			**1**	**2**	**3**	**4**	**5**	**6**	**7**	**8**	**9**	**10**
1. Age in years	69.20	10.94										
2. Felt age	4.37	1.73	0.11^***^									
3. Care recipient age	76.07	8.12	0.34^***^	0.10^***^								
4. Age difference between caregiver and care recipient	6.87	11.18	−0.73^***^	−0.04	0.39^***^							
5. Sex	1.69	0.46	−0.24^***^	−0.14^***^	0.08^**^	0.29^***^						
6. Education	2.60	1.09	−0.17^***^	0.13^***^	0.04	0.19^***^	−0.08^**^					
7. Hours of caregiving per day	1.18	0.76	0.08^**^	−0.11^***^	0.01	−0.08^*^	0.02	−0.12^***^				
8. Number of health conditions	1.35	1.42	0.17^***^	−0.16^***^	0.03	−0.15^***^	−0.02	−0.13^***^	0.12^***^			
9. Depression	7.17	7.94	−0.12^***^	−0.33^***^	−0.04	0.08^**^	0.20^***^	−0.04	0.13^***^	0.15^***^		
10. Stress	19.25	9.83	−0.07^*^	−0.30^***^	−0.02	0.05	0.19^***^	0.03	0.36^***^	0.08^**^	0.54^***^	
11. Positive aspects of caregiving	28.27	28.27	0.01	0.06^*^	0.07^*^	0.04	−0.14^***^	−0.14^***^	0.06	0.04	−0.17^***^	0.24^***^

^***^p values < 0.001. ^**^p values < 0.01 ^*^p values < 0.05. *p* values significant after Bonferroni's correction < 0.001. Correlation coefficients ≤ 0.09 were considered negligible, between 0.10 and 0.29 were considered small, between 0.30 and 0.49 were considered moderate, and ≥0.50 were considered large (Cohen, 1988). SD, Standard deviation.

### 3.2 Change in caregivers' FA

FA ICCs was 0.81 (95% CI: 0.78; 0.83). Spousal caregivers' mean FA at baseline was 4.36 (*SD* = 1.72), and results of multilevel modeling suggested that their FA did not significantly change over the two-year study period (adjusted for age, sex, and education mean slope = −0.02; 95% CI: −0.08; 0.04). Adult-child caregivers' mean FA at baseline was 4.45 (*SD* = 1.77), and results of multilevel modeling suggested their FA did not significantly change over the 2-year study period (adjusted for age, sex, and education mean slope = −0.07; 95% CI: −0.22; 0.08).

#### 3.2.1 FA at baseline as predictor of health and wellbeing over time

ICCs for measures of health and wellbeing were as follows: number of health conditions = 0.94 (95% CI: 0.93; 0.94); depression = 0.83 (95% CI: 0.81; 0.85); stress = 0.87 (95% CI: 0.85; 0.89); and positive aspects of caregiving = 0.88 (95% CI: 0.86; 0.89) between average measurements. Multilevel models exploring FA at baseline as a predictor of health and wellbeing showed that, among spousal caregivers, depression slightly increased over the 2-year study period (1.19, 95% CI: 0.89; 1.49). Younger FA was associated with lower depression at baseline (−1.42, 95% CI: −1.62; −1.23), but was not associated with the rate of change in depression over time. The number of spousal caregivers' health conditions also increased over the course of the study (1.21, 95% CI: 0.1; 0.16; 1.26). Younger FA was associated with fewer health conditions at baseline (0.96, 95% CI: 0.94; 0.99) but was not associated with the rate of change in health conditions over time. Spousal caregivers' stress increased over the course of the study (2.69, 95% CI: 2.40; 2.99). Younger FA was associated with spousal caregivers' lower stress at baseline (−1.19, 95% CI: −1.19; −0.90), but it did not contribute to the rate of change in stress over time. Finally, positive aspects of caregiving did not change significantly over the 2-year study period. Younger FA was associated with more positive aspects of caregiving at baseline (0.18, 95% CI: 0.01; 0.35), but did not contribute to any change in positive aspects of caregiving over time. Full model results, with estimates for covariates, are available in Supplementary Tables 5–8.

Among adult-child caregivers, depression slightly increased over the 2-year study period (0.95, 95% CI: 0.22; 1.68). Younger FA was associated with lower depression at baseline (−1.78, 95% CI: −2.28; −1.27), but was not associated with the rate of change in depression over time. The number of adult-child caregivers' health conditions also increased over the course of the study (1.16, 95% CI: 0.1.01; 1.34). Younger FA was associated with fewer health conditions at baseline (0.90, 95% CI: 0.83; 0.99) but was not associated with the rate of change in health conditions over time. Adult-child caregivers' stress increased over the course of the study (1.36, 95% CI: 0.54; 2.19). Younger FA was associated with adult-child caregivers' lower stress at baseline (−1.56, 95% CI: −2.05; −1.07), but it did not contribute to the rate of change in stress over time. Finally, positive aspects of caregiving did not change significantly over the 2-year study period. Younger FA was associated with more positive aspects of caregiving at baseline (0.44, 95% CI: 0.08; 0.81), but did not contribute to any change in positive aspects of caregiving over time. Full model results, with estimates for covariates, are available in Supplementary Tables 9–12.

#### 3.2.2 Hours of caregiving per day as moderator

In spousal caregivers, but not in adult-child caregivers, the interaction between FA at baseline and hours of caregiving per day at baseline explained a significant amount of variance in depression at baseline and over time. More specifically, a younger FA was more strongly associated with lower depression at baseline (−0.37, 95% CI: −0.61; −0.14) and over time (−0.27, 95% CI: −0.52; −0.02) among spousal caregivers providing more hours of caregiving per day. The interaction between FA at baseline and hours of caregiving per day at baseline did not explain a significant amount of variance in number of health conditions, stress, and positive aspects of caregiving at baseline for spousal caregivers (*p* > 0.05) nor adult-child caregivers (*p* > 0.05) over time. Full results of the moderation models, with estimates for covariates, are available in Supplementary Tables 6–13.

### 3.3 Bidirectional influences between FA and health and wellbeing

**Figure 1:**
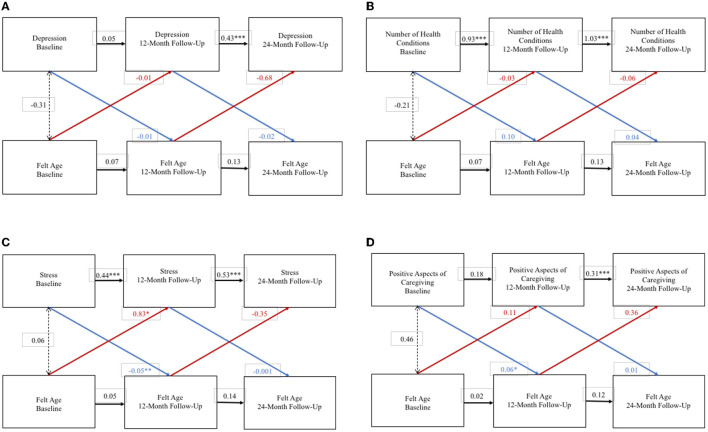
Random intercept cross lagged panel models – spousal caregivers. **(A)** Depression. **(B)** Number of health conditions. **(C)** Stress. **(D)** Positive aspects of caregiving. Statistics are standardized regression coefficients. Each association adjusted for age, sex, and education. CFI, Comparative Fit Index; RMSEA, Root Mean Square Error of Approximation; SRMR, Standardized Root Mean Square Residual; BIC, Bayesian Information Criterion. *p < 0.05, ** *p* < 0.01, ***p < 0.001.

For spousal caregivers, WP FA and WP depression did not exert an effect on one another over the 2-year study period (Figure 1A). But, at the BP level, an older FA was associated with higher depression. WP FA and WP number of health conditions also did not exert an effect on each other over the 2-year study period (Figure 1B), nor were they associated at the BP level. An older WP FA at baseline was associated with greater WP stress at 1-year follow-up, and higher WP stress at baseline was associated with older WP FA at 1-year follow-up (Figure 1C). Moreover, at the BP level, an older FA was associated with greater stress. Finally, higher WP positive aspects of caregiving at baseline was associated with a younger FA at 1-year follow-up (Figure 1D), but at the BP level, FA was not significantly associated with positive aspects of caregiving.

**Figure 2:**
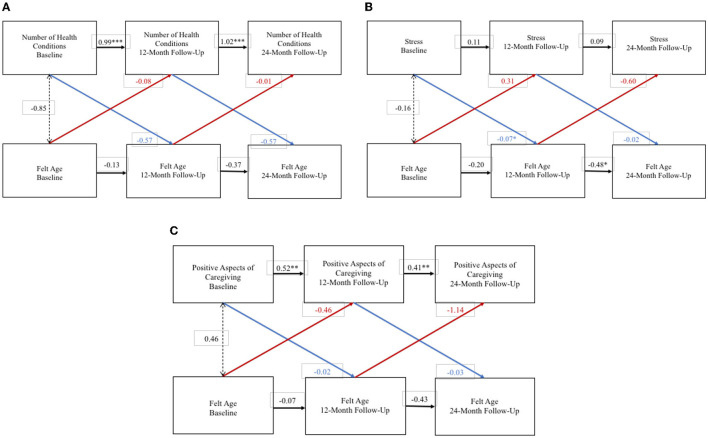
Random intercept cross lagged panel models—adult-child caregivers. **(A)** Number of health conditions. **(B)** Stress. **(C)** Positive aspects of caregiving. Statistics are standardized regression coefficients. Each association adjusted for age, sex, and education. CFI, Comparative Fit Index; RMSEA, Root Mean Square Error of Approximation; SRMR, Standardized Root Mean Square Residual; BIC, Bayesian Information Criterion. *p < 0.05, ** *p* < 0.01, ***p < 0.001.

For adult-child caregivers the RI-CLPM exploring bidirectional effects between FA and depression did not converge. WP FA and WP number of health conditions did not exert an effect on each other over the 2-year study period (Figure 2A), and FA and health conditions were not significantly associated at the BP level. WP FA and WP stress did not exert an effect on each other over the 2-year study period (Figure 2B), but an older FA was associated with greater stress at the BP level. Although the association between WP FA and WP stress was of larger size than the BP association, there was less statistical confidence in the WP results. Finally, WP FA and WP positive aspects of caregiving did not exert a significant effect on each other over the 2-year study period (Figure 2C), and FA and positive aspects of caregiving were not significantly associated at the BP level.

## 4 Discussion

In this study, we analyzed FA among family caregivers of people with dementia, hypothesizing that caregivers' health and wellbeing would both shape and be shaped by caregivers' FA, and that the nature of caregiving (i.e., hours of caregiving provided per day) would moderate the cross-sectional association between FA and caregivers' health and wellbeing. In so doing, we conducted the first study to explore FA among family caregivers of people with dementia, which is a key addition to the emerging scholarship on caregivers' subjective aging experiences and the broader literature on the connections between family relationships and VoA. Indeed, given the number of people caring for older family members, and the extent to which caregiving impacts both daily life and longer-term health and wellbeing outcomes, caregiving is a critically relevant activity through which to explore how VoA concepts develop, and whether caregivers should be the target of interventions to improve their VoA.

Among our sample, the mean level FA for adult-children was older than that for spousal caregivers. That is, adult-child caregivers felt older to a greater extent than spousal caregivers. Though we were unable to directly compare caregivers to non-caregivers, the percentages of spousal caregivers and adult-child caregivers with older FAs in this study were both more than double those in the general UK population aged 50+ reported in other studies (10.2% in another UK sample; 13), suggesting that there may be something about the caregiving role that makes people more likely to feel older than they are. Notably, however, many caregivers felt younger than or the same as their chronological age, which seemed to offer a resource for their health and wellbeing. Indeed, a younger FA was associated at the cross-sectional level with various positive health and wellbeing indicators in both spousal and adult-child caregivers.

Further, on average, FA did not change over the 2-year study period for either spousal caregivers or adult-child caregivers. This finding contributes to the growing literature exploring within-person variation in VoA constructs (Cohn-Schwartz et al., 2022). Existing studies have analyzed within-person changes in FA at various time intervals, for example within a single day (Kornadt et al., 2021), between days (O'Brien and Smyth, 2023), over 4 years (Kornadt et al., 2016) and over 10 years (Ward, 2013; Hughes and Lachman, 2018). These studies typically conclude that a greater percentage of the variance in FA is attributed to individual, between-person level differences rather than to within-person fluctuations over time. More research is required to fully understand the rate at which FA changes over time and how contextual factors, such as caregiving, contribute to the rate of change at various time intervals. For now, however, as it pertains to caregivers, even though caregivers (and especially spousal caregivers) may be more likely to have an older FA compared to non-caregivers, our study suggests they may not develop even older FAs over time. Although there is not empirical evidence on this, it is possible that caregivers' FA rapidly changes before and after assuming the caregiving role, but that, over time, caregivers are resilient in their adaptation to the caregiving role, which does not place a further negative impact on how they appraise their own aging. However, this speculation requires further empirical testing.

### 4.1 Connections between caregivers' FA and health and wellbeing: key findings

#### 4.1.1 Depression

Among spousal caregivers, a younger FA at baseline was associated with lower depression at baseline and a lower increase in depression over time. Moreover, hours of care provided per day moderated the association between spousal caregivers' FA and depression; the more hours of care a spousal caregiver provided, the stronger the association between their younger FA and lower depression. This finding offers evidence in support of our hypothesis that the nature of caregiving may not only impact FA, but also FA's connection to caregivers' health and wellbeing. Specifically, they highlight the potential for FA to be a protective factor for spousal caregivers' depression, especially in spousal caregivers who are caring for more hours per day.

However, we were surprised that the direction of the moderation was such that more caregiving hours were associated with a stronger correlation between younger FA and less depression. Perhaps this finding in our study reflects the findings in existing literature whereby more hours of care provided is highly associated with more subjective caregiving gains (Quinn et al., 2012). It is possible that more hours of care, then, may be a protective factor that “boosts” the value of a younger FA.

It was also interesting that hours of care per day was a moderator of FA and depression for spousal caregivers but not adult-child caregivers. Adult-child dementia caregivers are typically more likely to experience depressive symptoms than spousal dementia caregivers (Watson et al., 2019). However, in our sample, though adult-child caregivers had higher mean-level depressive symptoms than spousal caregivers, the differences were not significant. Thus, it is possible that our findings in this study are reflective of the peculiarities of this sample and are not generalizable to caregivers in general.

Nonetheless, given the high number of dementia family caregivers who experience depression (Mahoney et al., 2005; Joling et al., 2010; Wulff et al., 2020), and the many interventions designed to support caregivers' mental health (Hopkinson et al., 2019), our findings about the connections between caregivers' FA and their depression are pertinent and may offer insight into a psychosocial mechanism that can be incorporated into caregiver interventions (Mahoney et al., 2005; Joling et al., 2010; Wulff et al., 2020).

#### 4.1.2 Number of health conditions

On average, dementia caregivers have more chronic health conditions than non-dementia caregivers (Alzheimer's Association, 2022) and non-caregivers (Janson et al., 2022), and health conditions (Sabatini et al., 2022, 2023b), especially the onset of new diagnoses (Rupprecht et al., 2022), are associated with poorer VoA. As such, we were surprised that the caregivers in our sample did not experience changes in FA when they experienced changes to the number of health conditions they had. Indeed, among both spousal and adult-child caregivers, FA was associated with the number of health conditions at a cross-sectional level only. The number of health conditions experienced by caregivers in this sample changed minimally over the course of the study, which may have contributed to our null findings. Dementia caregivers often have unmet healthcare needs (Waligora et al., 2019) and many report missing appointments with their own healthcare providers (Wang et al., 2015). It is possible that the caregivers in our sample had health conditions that were not diagnosed and, thus, were not reported in the survey. Ultimately, we encourage future studies on the connections between VoA and caregivers' health conditions to utilize samples of caregivers who have experienced changes in diagnoses over time.

#### 4.1.3 Stress

A major study finding regarding stress was that, in the bi-directional RI-CLPMs, stress at baseline predicted spousal caregivers' FA 12 months later. Caregivers often experience high levels of stress, and alleviating caregiver stress is a typical priority of caregiver interventions (Gilhooly et al., 2016). Our study's finding that caregiver stress predicts worsening FA up to a year later offers another justification in support of efforts to reduce caregivers' stress. Further, one mechanism through which caregiver stress leads to poorer health outcomes may be through FA, and we urge future research in this regard.

#### 4.1.4 Positive aspects of caregiving

Researchers have long understood that, alongside the negative subjective appraisals of caregiving, caregivers often also report positive subjective appraisals of caregiving (Zarit, 2012). These positive appraisals of caregiving are associated with personal benefits (such as increased sense of purpose) that can last even after caregiving has ended (Bangerter et al., 2019). Positive appraisals of caregiving can also buffer the consequential impacts of negative caregiving experiences (Rapp and Chao, 2000). Thus, it is helpful to know what psychosocial factors contribute to caregivers feeling more positively about their caregiving. Also, analyzing how positive aspects of caregiving are associated with FA can offer an indication of how caregivers' subjective caregiving experiences impact their FA.

In this study, a younger FA was cross-sectionally associated with higher positive aspects of caregiving for both spousal and adult-child caregivers. Among spousal caregivers, higher positive aspects of caregiving at baseline were associated with younger FA 1 year later. This finding supports our hypothesis that the nature of caregiving may impact caregivers' FA. Notably, as with depression and stress, the connections between FA and the positive appraisals of caregiving were present for spousal caregiver but not adult-child caregivers. It is plausible that these differences are a product of age differences. Indeed, existing research suggests that age is a moderator of the association between health and FA (Prasad et al., 2022).

### 4.2 Limitations

There are a few limitations to our study, mostly related to measurement of certain variables. First, though we used a measure of FA that is used in previous studies and that is recommended for use in samples with large age ranges (Montepare, 2020), we asked participants to solely report how old they feel in general and not in relation to different domains of their life. Knowing the dimension-specific FA could have offered insight into differences between spousal and adult-child caregivers FA. However, even though several dimensions of FA such as look age, do age, and interest age have been proposed (Kastenbaum et al., 1972), at a statistical level these dimensions load onto a single factor supporting a unidimensional construct (Barak, 1987; Hubley and Russell, 2009; Teuscher, 2009). Second, another limitation of our study's measures is that hours of caregiving per day were assessed with an ordinal variable. It was therefore not possible to capture the full range of hours of caregiving per day that caregivers provided, which may have impacted on our analyses exploring the moderating role of hours of caregiving per day in the associations of FA with the health and wellbeing correlates investigated. Including hours of caregiving per day as a continuous variable in future studies is warranted. Third, most available studies explore FA in WEIRD (western, educated, industrialized, rich, and democratic) countries, and in line with this we explored FA in United Kingdom residents who were almost all of White ethnicity. This limits generalizability of our study results. Fourth, as in many studies following people with dementia and their caregivers over time, a significant proportion of participants withdrew from the study over the 2-year period. Compared to caregivers who stayed in the study, those who withdrew from the study, on average, felt older and scored more negatively on health and wellbeing indicators at baseline. This may have impacted on our results that found no effect of FA and health and wellbeing over time. Nonetheless, in the IDEAL study the main reason for caregivers interrupting their participation in the study was generally due to the person with dementia withdrawing from the study, moving into residential care, or dying. Fifth, in the current study we focused on time spent caregiving, but other variables that measure caregiving intensity, such as the amount of physical difficulty from caregiving, may moderate the associations of FA with health and wellbeing in ways that hours of care per day does not. Sixth, in this study measures were administered in the same order to all participants. This is a possible limitation of the study as it could bias the results due to fatigue or learning effects.

Despite these limitations, this is one of the first studies using longitudinal data to explore FA in caregivers of people with dementia. Moreover, the longitudinal study design made it possible to explore whether FA and health and wellbeing constructs have an effect on each other between 1 year and the next. This is a strength as the causal effects of FA and health and wellbeing variables are not well understood.

## 5 Conclusions

This study is a fundamental first step in the scholarship on caregiving and FA, and extends the literature on how family relationships may impact FA. A high proportion of caregivers in this sample felt as old as or older than their age, which could be a risk factor for poorer future health. Indeed, at least among spousal caregivers, FA was associated with higher stress 1 year later, which can in turn lead to poorer health. Also among spousal caregivers, hours of care per day moderated the association between FA and stress, further reinforcing the nature of caregiving as a contextual factor that not only impacts FA, but also impacts FA's relationship with variables that can in turn influence health and wellbeing. Ultimately, we urge continued research on the connections between caregiving and FA, and how interventions might support caregivers' positive VoA. Doing so will help translate VoA scholarship in ways that can meaningfully improve caregivers' lives.

## Data availability statement

The original contributions presented in the study are included in the article/Supplementary material, further inquiries can be directed to the corresponding author.

## Ethics statement

IDEAL was approved by the Wales 5 Research Ethics Committee (reference: 13/WA/0405) and the Ethics Committee of the School of Psychology, Bangor University (reference: 2014-11684). IDEAL is registered with UK Clinical Research Network, registration No. 16593. The studies were conducted in accordance with the local legislation and institutional requirements. Written informed consent for participation in this study was provided by the participants' legal guardians/next of kin.

## Author contributions

SS: Conceptualization, Data analysis, Writing–original draft. ST: Conceptualization, Writing–original draft. RM: Funding acquisition, Writing–review & editing. CO: Writing–review & editing. JT: Writing–review & editing. AH: Writing–review & editing. LA: Writing–review & editing. CP: Writing–review & editing. LC: Conceptualization, Funding acquisition, Writing–review & editing.
