# Supplementary material for: Correlates of felt age in caregivers of people with dementia: findings from the IDEAL study
Source: Front Psychol. 2024 Jan 12;14:1287842. doi: 10.3389/fpsyg.2023.1287842 (PMC10811155; doi:10.3389/fpsyg.2023.1287842)
Supplement: Supplementary file 1 [file Data_Sheet_1.docx]

Supplementary Material

| **Supplementary Table 1**  *Sociodemographic Characteristics at Baseline of Participants who Stayed in the Study at One-year Follow-up and who Dropped Out at One-year Follow-up.* | | | |
| --- | --- | --- | --- |
| Variables | Participants who stayed in the study at one-year follow-up  (N= 939) | Participants who dropped out at one-year follow-up  (N= 264) | p-value |
| Age in years, Mean (SD; range) | 69.28 (10.50) | 68.89 (12.34) | 0.612 |
| Sex: Women, n (%) | 646 (68.8) | 181 (68.6) | 0.942 |
| Educational level, Mean (SD) | 2.62 (1.09) | 2.51 (1.08) | 0.127 |
| Missing, n | 6 |  |  |
| Ethnicity, n (%) |  |  |  |
| White British | 908 (96.7) | 251 (95.1) | 0.500 |
| White other | 23 (2.4) | 9 (3.4) |  |
| Other | 8 (0.9) | 4 (1.5) |  |
| Hours of caregiving per day, Mean (SD) | 1.15 (0.75) | 1.26 (0.77) | 0.039 |
| Missing, n | 11 |  |  |
| Felt age, Mean (SD) | 4.41 (1.70) | 4.23 (1.82) | 0.122 |
| Missing, n | 6 |  |  |
| Depression, Mean (SD) | 6.95 (7.68) | 7.97 (8.80) | 0.076 |
| Missing | 57 |  |  |
| Number of health conditions, Mean (SD) | 1.40 (1.42) | 1.18 (1.39) | 0.039 |
| Missing, n | 66 |  |  |
| Stress, Mean (SD) | 18.75 (9.57) | 21.09 (10.54) | 0.001 |
| Missing, n | 58 |  |  |
| Positive aspects of caregiving, Mean (SD) | 28.10 (7.35) | 28.87 (7.50) | 0.141 |
| Missing, n | 26 |  |  |
| Notes:  N= number.  SD= Standard deviation.  p-values calculated using chi-squared tests for categorical variables and analysis of variance (ANOVA) for continuous variables.  *For analyses purposes hours of caregiving per day was treated as an ordinal variable and in the current table descriptive statistics are reported for the hours of caregiving as an ordinal variable. | | | |

| **Supplementary Table 2**  *Sociodemographic Characteristics at Baseline of Participants who Stayed in the Study between One and Two-year Follow-up and who Dropped Out between One and Two-year Follow-up.* | | | |
| --- | --- | --- | --- |
| Variables | Participants who stayed in the study between one and two-year follow-up  (N= 701) | Participants who dropped out between one and two-year follow-up  (N= 238) | p-value |
| Age in years, Mean (SD; range) | 69.01 (10.35) | 70.08 (10.95) | 0.177 |
| Sex: Women, n (%) | 476 (67.9) | 170 (71.4) | 0.310 |
| Educational level, Mean (SD) | 2.65 (1.10) | 2.56 (1.09) | 0.278 |
| Missing, n | 4 |  |  |
| Ethnicity, n (%) |  |  |  |
| White British | 677 (96.6) | 231 (97.1) | 0.497 |
| White other | 19 (2.7) | 4 (1.68) |  |
| Other | 5 (0.7) | 3 (1.3) |  |
| Hours of caregiving per day, Mean (SD) | 1.10 (0.76) | 1.32 (0.70) | 0.0001 |
| Missing, n | 10 |  |  |
| Felt age, Mean (SD) | 4.46 (1.68) | 4.27 (1.75) | 0.144 |
| Missing, n | 6 |  |  |
| Depression, Mean (SD) | 6.72 (7.47) | 7.64 (8.25) | 0.117 |
| Missing | 37 |  |  |
| Number of health conditions, Mean (SD) | 1.37 (1.41) | 1.48 (1.45) | 0.307 |
| Missing, n | 29 |  |  |
| Stress, Mean (SD) | 18.08 (9.37) | 20.77 (9.87) | 0.002 |
| Missing, n | 41 |  |  |
| Positive aspects of caregiving, Mean (SD) | 27.90 (7.20) | 28.70 (7.75) | 0.150 |
| Missing, n | 15 |  |  |
| Notes:  N= number.  SD= Standard deviation.  p-values calculated using chi-squared tests for categorical variables and analysis of variance (ANOVA) for continuous variables.  *For analyses purposes hours of caregiving per day was treated as an ordinal variable and in the current table descriptive statistics are reported for the hours of caregiving as an ordinal variable. | | | |

| **Supplementary Table 3**  *Correlation Matrix of Continuous Study Variables at Baseline for the Subsamples of Spousal Caregivers and Adult-Child Caregivers* | | | | | | | | | | | | |
| --- | --- | --- | --- | --- | --- | --- | --- | --- | --- | --- | --- | --- |
| **Spousal Caregivers** | | | | | | | | | | | | |
|  | Mean | SD | Pearson’s *r* correlation coefficient | | | | | | | | | |
|  |  |  | 1 | 2 | 3 | 4 | 5 | 6 | 7 | 8 | 9 | 10 |
| 1. Age in years | 72.37 | 8.22 |  |  |  |  |  |  |  |  |  |  |
| 2. Felt age | 4.36 | 1.72 | .16*** |  |  |  |  |  |  |  |  |  |
| 3. Care recipient age | 75.00 | 7.80 | .78*** | .09* |  |  |  |  |  |  |  |  |
| 4. Age difference between caregiver and care recipient | 2.62 | 5.34 | -.40*** | -.12*** | .26*** |  |  |  |  |  |  |  |
| 5. Sex | 1.67 | .47 | -.24*** | -.14*** | .07* | .47*** |  |  |  |  |  |  |
| 6. Education | 2.51 | 1.10 | -.07* | .12*** | -.06 | .03 | -.11*** |  |  |  |  |  |
| 7. Hours of caregiving per day | 1.23 | .77 | -.04 | -.11*** | .05 | .14*** | .05 | -.10** |  |  |  |  |
| 8. Number of health conditions | 1.46 | 1.43 | .10* | -.15*** | .11*** | .01 | -.01 | -.11*** | .11*** |  |  |  |
| 9. Depression | 7 | 7.55 | -.12*** | -.31*** | -.05 | .12*** | .21*** | -.05 | .15*** | .16*** |  |  |
| 10. Stress | 19.33 | 9.77 | -.13** | .37*** | -.03 | .15*** | .21*** | .03 | .38*** | -.07* | -.55*** |  |
| 11. Positive aspects of caregiving | 28.01 | 7.45 | .14*** | .05 | .08** | -.10** | -.16*** | -.17*** | .07* | .07* | -.18*** | .25*** |
| *Note.* Correlation coefficients ≤ .09 were considered negligible, between .10 and .29 were considered small, between .30 and .49 were considered moderate, and ≥ .50 were considered large (63).  SD = Standard deviation.  **p* < .05. ***p* < .01. ****p* < .001. *p values significant after Bonferroni’s correction <.001.* | | | | | | | | | | | | |
| **Supplementary Table 3 (continued)**  *Correlation Matrix of Continuous Study Variables at Baseline for the Subsamples of Spousal Caregivers and Adult-Child Caregivers* | | | | | | | | | | | | |
| **Adult-Child Caregivers** | | | | | | | | | | | | |
|  | Mean | SD | Pearson’s *r* correlation coefficient | | | | | | | | | |
|  |  |  | 1 | 2 | 3 | 4 | 5 | 6 | 7 | 8 | 9 | 10 |
| 1. Age in years | 52.98 | 8.42 |  |  |  |  |  |  |  |  |  |  |
| 2. Felt age | 4.4 | 1.77 | .17* |  |  |  |  |  |  |  |  |  |
| 3. Care recipient age | 81.55 | 7.47 | .58*** | .13 |  |  |  |  |  |  |  |  |
| 4. Age difference between caregiver and care recipient | 28.57 | 7.34 | -.56*** | -.06 | .35*** |  |  |  |  |  |  |  |
| 5. Sex | 1.80 | .40 | -.14 | -.13 | -.09 | .07 |  |  |  |  |  |  |
| 6. Education | 3.09 | .88 | .09 | .17* | .25*** | .15* | -.12 |  |  |  |  |  |
| 7. Hours of caregiving per day | .92 | .65 | .06 | -.09 | .13 | .07 | -.10 | .01 |  |  |  |  |
| 8. Number of health conditions | .82 | 1.26 | -.004 | -.22** | -.06 | -.06 | .07 | -.01 | .03 |  |  |  |
| 9. Depression | 8.02 | 9.62 | -.11 | -.44*** | -.12 | .001 | .13 | -.06 | .10 | .18* |  |  |
| 10. Stress | 18.88 | 10.13 | -.02 | -.38*** | .09 | .11 | .03 | .08 | .29*** | .15* | .52*** |  |
| 11. Positive aspects of caregiving | 29.56 | 6.90 | -.22** | .13 | .14 | .11 | -.09 | -.13 | .09 | -.04 | -.19** | .20** |
| *Notes.* Correlation coefficients ≤ .09 were considered negligible, between .10 and .29 were considered small, between .30 and .49 were considered moderate, and ≥ .50 were considered large (63).  SD= Standard deviation.  **p* < .05. ***p* < .01. ****p* < .001. *p values significant after Bonferroni’s correction <.001.* | | | | | | | | | | | | |

| **Supplementary Table 4**  *Correlation Matrix of Continuous Study Variables at One-Year Follow-up for the Subsamples of Spousal Caregivers and Adult-Child Caregivers.* | | | | | | | | | | | | |
| --- | --- | --- | --- | --- | --- | --- | --- | --- | --- | --- | --- | --- |
| **Spousal Caregivers** | | | | | | | | | | | | |
|  | Mean | SD | Pearson’s *r* correlation coefficient | | | | | | | | | |
|  |  |  | 1 | 2 | 3 | 4 | 5 | 6 | 7 | 8 | 9 | 10 |
| 1. Age in years | 73.19 | 7.93 |  |  |  |  |  |  |  |  |  |  |
| 2. Felt age | 4.41 | 1.71 | .20*** |  |  |  |  |  |  |  |  |  |
| 3. Care recipient age | 75.77 | 7.62 | .77*** | -10** |  |  |  |  |  |  |  |  |
| 4. Age difference between caregiver and care recipient | 2.57 | 5.31 | -.39*** | -.15*** | .29*** |  |  |  |  |  |  |  |
| 5. Sex | 1.66 | .47 | -.25*** | -.19*** | .07* | .47*** |  |  |  |  |  |  |
| 6. Education | 2.54 | 1.10 | -.08* | .13*** | -.08* | .03 | -.11** |  |  |  |  |  |
| 7. Hours of caregiving per day | 1.33 | .73 | -.02 | -.22*** | .06 | .11** | .10** | -.07 |  |  |  |  |
| 8. Number of health conditions | 1.85 | 1.66 | .13*** | -.09* | .15*** | .02 | -.003 | -.14*** | .09* |  |  |  |
| 9. Depression | 8.32 | 9.0 | -.15*** | -.43*** | -.05 | .15*** | .24*** | -.08* | .16*** | .26*** |  |  |
| 10. Stress | 22.17 | 10.10 | -.12** | -.41*** | .01 | .19*** | .26*** | -.01 | .41*** | .11** | .55*** |  |
| 11. Positive aspects of caregiving | 27.72 | 7.75 | .17*** | .11** | .09* | -.13** | .19*** | -.12** | .02 | .05 | -.19*** | .32*** |
| **Adult-Child Caregivers** | | | | | | | | | | | | |
|  | Mean | SD | Pearson’s *r* correlation coefficient | | | | | | | | | |
|  |  |  | 1 | 2 | 3 | 4 | 5 | 6 | 7 | 8 | 9 | 10 |
| 1. Age in years | 53.67 | 8.05 |  |  |  |  |  |  |  |  |  |  |
| 2. Felt age | 4.14 | 1.80 | .31*** |  |  |  |  |  |  |  |  |  |
| 3. Care recipient age | 82.76 | 7.08 | .71*** | .33*** |  |  |  |  |  |  |  |  |
| 4. Age difference between caregiver and care recipient | 29.10 | 5.86 | -.52*** | -.03 | .24** |  |  |  |  |  |  |  |
| 5. Sex | 1.83 | .38 | -.07 | -.04 | -.01 | .07 |  |  |  |  |  |  |
| 6. Education | 3.16 | .89 | .19* | .17 | .24** | .15 | -.12 |  |  |  |  |  |
| 7. Hours of caregiving per day | .98 | .66 | -.09 | -.07 | -.02 | .11 | -.04 | .01 |  |  |  |  |
| 8. Number of health conditions | 1.05 | 1.44 | .09 | -.16 | .003 | -.13 | .05 | -.02 | .14 |  |  |  |
| 9. Depression | 9.73 | 10.92 | -.29** | -.53*** | -.27** | .09 | .07 | -.04 | .11 | .28** |  |  |
| 10. Stress | 20.08 | 9.13 | -.04 | -.48*** | .05 | .13 | .06 | .07 | .25** | .23** | .54*** |  |
| 11. Positive aspects of caregiving | 30.05 | 7.42 | -.06 | .03 | -.02 | .07 | -.17 | -.11 | .08 | -.05 | -.18* | -.25* |
| *Notes.* Correlation coefficients ≤ .09 were considered negligible, between .10 and .29 were considered small, between .30 and .49 were considered moderate, and ≥ .50 were considered large (63). SD = Standard deviation.  **p* < .05. ***p* < .01. ****p* < .001. *p values significant after Bonferroni’s correction <.001.* | | | | | | | | | | | | |

| **Supplementary Table 5**  *Correlation Matrix of Continuous Study Variables at Two-Year Follow-up for the Subsamples of Spousal Caregivers and Adult-child Caregivers* | | | | | | | | | | | | |
| --- | --- | --- | --- | --- | --- | --- | --- | --- | --- | --- | --- | --- |
| **Spousal Caregivers** | | | | | | | | | | | | |
|  | Mean | SD | Pearson’s *r* correlation coefficient | | | | | | | | | |
|  |  |  | 1 | 2 | 3 | 4 | 5 | 6 | 7 | 8 | 9 | 10 |
| 1. Age in years | 73.76 | 7.85 |  |  |  |  |  |  |  |  |  |  |
| 2. Felt age | 4.39 | 1.71 | .21*** |  |  |  |  |  |  |  |  |  |
| 3. Care recipient age | 76.24 | 7.74 | .78*** | .11** |  |  |  |  |  |  |  |  |
| 4. Age difference between caregiver and care recipient | 2.48 | 5.18 | -.35*** | -.14*** | .31*** |  |  |  |  |  |  |  |
| 5. Sex | 1.65 | .48 | -.22*** | -.19*** | .09* | .47*** |  |  |  |  |  |  |
| 6. Education | 2.57 | 1.10 | -.06 | .14*** | -.08* | -.03 | -.13** |  |  |  |  |  |
| 7. Hours of caregiving per day | 1.38 | .70 | .03 | -.17*** | .06 | .05 | .02 | -.10* |  |  |  |  |
| 8. Number of health conditions | 2.07 | 1.85 | .17*** | -.04 | .21*** | .05 | .05 | -.11** | .05 |  |  |  |
| 9. Depression | 8.80 | 9.16 | -.13** | -.40*** | -.03 | .13*** | .21*** | -.08* | .18*** | .14*** |  |  |
| 10. Stress | 23.29 | 10.13 | -.13** | -.36*** | .002 | .19*** | .25*** | -.01 | .42*** | .09* | .55*** |  |
| 11. Positive aspects of caregiving | 27.73 | 7.82 | .10* | .07 | .04 | -.09* | -.18*** | -.09* | -.01 | .04 | -.24*** | .32*** |
| **Adult-Child Caregivers** | | | | | | | | | | | | |
|  | Mean | SD | Pearson’s *r* correlation coefficient | | | | | | | | | |
|  |  |  | 1 | 2 | 3 | 4 | 5 | 6 | 7 | 8 | 9 | 10 |
| 1. Age in years | 54.25 | 8.0 |  |  |  |  |  |  |  |  |  |  |
| 2. Felt age | 4.32 | 1.66 | .24* |  |  |  |  |  |  |  |  |  |
| 3. Care recipient age | 83.07 | 7.28 | .72*** | .26* |  |  |  |  |  |  |  |  |
| 4. Age difference between caregiver and care recipient | 28.82 | 5.82 | -.49*** | -.01 | .26** |  |  |  |  |  |  |  |
| 5. Sex | 1.83 | .37 | .02 | -.24* | .05 | .03 |  |  |  |  |  |  |
| 6. Education | 3.18 | .89 | .17 | .37*** | .25* | .06 | -.10 |  |  |  |  |  |
| 7. Hours of caregiving per day | .91 | .67 | -.16 | -.20 | -.10 | .11 | .11 | -.10 |  |  |  |  |
| 8. Number of health conditions | .99 | 1.34 | .04 | -.13 | -.004 | -.07 | -.004 | -.03 | -.10 |  |  |  |
| 9. Depression | 9.25 | 11.95 | -.22* | -.39*** | -.15 | .14 | .14 | -.18 | .25* | .22* |  |  |
| 10. Stress | 21.75 | 10.15 | .001 | -.36*** | -.03 | -.04 | .14 | -.09 | .39*** | .24* | .49*** |  |
| 11. Positive aspects of caregiving | 29.41 | 6.88 | -.15 | .09 | -.10 | .09 | -.24* | -.03 | .01 | -.003 | -.11 | .23* |
| *Notes.* Correlation coefficients ≤ .09 were considered negligible, between .10 and .29 were considered small, between .30 and .49 were considered moderate, and ≥ .50 were considered large (63). **p* < .05. ***p* < .01. ****p* < .001. *p values significant after Bonferroni’s correction <.001.* | | | | | | | | | | | | |

| **Supplementary Table 6**  *Association of Felt Age and Depression Over Time, and Moderating Role of Hours of Caregiving per Day in Spousal Caregivers* | | |
| --- | --- | --- |
|  | **Depression Over Time** | |
|  | Mean intercept  (Estimate, 95% CI); p-value | Mean slope  (Estimate, 95% CI); p-value |
|  | 7.19 (5.74; 8.63); <.001 | 1.19 (.89; 1.49); <.001 |
| Age | -.09 (-.15; -.04); .001 |  |
| Sex | 3.31 (2.32; 4.31); <.001 |  |
| Education | -.32 (-.74; .09); .104 |  |
|  | **Felt Age as a Predictor of Depression Over Time** | |
|  | Mean intercept  (Estimate, 95% CI); p-value | Mean slope  (Estimate, 95% CI); p-value |
| Felt age | -1.42 (-1.62; -1.23); <.001 | -.32 (-.49; -.14); <.001 |
| Age | -.06 (-.11; -.002); .042 |  |
| Sex | 2.85 (1.91; 3.79); <.001 |  |
| Education | -.10 (-.49; .29); .617 |  |
|  | **Interaction Between Felt Age and Hours of Caregiving Per Day as a Predictor of Depression Over Time** | |
|  | Mean intercept  (Estimate, 95% CI); p-value | Mean slope  (Estimate, 95% CI); p-value |
| Felt age | -.89 (-1.26; -.52); <.001 | .05 (-.32; .42); .789 |
| Hours of caregiving per day | 2.44 (1.29; 3.60); <.001 | 1.75 (.56; 2.94); .004 |
| Felt age x hours of caregiving per day | -.37 (-.61; -.14); .002 | -.27 (-.52; -.02); .032 |
| Age | -.05 (-.11; .003); .061 |  |
| Sex | 2.92 (1.97; 3.88); <.001 |  |
| Education | -.09 (-.49; .31); .643 |  |

| **Supplementary Table 7**  *Association of Felt Age and Number of Health Conditions Over Time, and Moderating Role of Hours of Caregiving per Day in Spousal Caregivers* | | |
| --- | --- | --- |
|  | **Number of Health Conditions Over Time** | |
|  | Mean intercept  (Incidence rate ratio, 95% CI); p-value | Mean slope  (Incidence rate ratio, 95% CI); p-value |
|  | 1.68 (1.39; 2.03); <.001 | 1.21 (1.16; 1.26); <.001 |
| Age | 1.02 (1.01; 2.80); <.001 |  |
| Sex | 1.02 (.89; 1.16); .814 |  |
| Education | .90 (.84; .94); <.001 |  |
|  | **Felt Age as a Predictor of Number of Health Conditions Over Time** | |
|  | Mean intercept  (Incidence rate ratio, 95% CI); p-value | Mean slope  (Incidence rate ratio, 95% CI); p-value |
| Felt age | .96 (.94; .99); .007 | 1.02 (.99; 1.04); .140 |
| Age | 1.02 (1.01; 1.03); <.001 |  |
| Sex | 1.00 (.88; 1.14); .981 |  |
| Education | .90 (.85; .95); <.001 |  |
|  | **Interaction Between Felt Age and Hours of Caregiving Per Day as Predictor of Number of Health Conditions Over Time** | |
|  | Mean intercept  (Incidence rate ratio, 95% CI); p-value | Mean slope  (Incidence rate ratio, 95% CI); p-value |
| Felt age | .97 (.92; 1.03); .305 | 1.04 (.99; 1.11); .120 |
| Hours of caregiving per day | 1.06 (.90; 1.26); .436 | 1.04 (.89; 1.23); .296 |
| Felt age x hours of caregiving per day | 1.00 (.96; 1.03); .801 | .98 (.94; 1.02); .801 |
| Age | 1.02 (1.01; 1.03); <.001 |  |
| Sex | 1.00 (.88; 1.14); .994 |  |
| Education | .90 (.86; .96); <.001 |  |

| **Supplementary Table 8**  *Association of Felt Age and Stress Over Time, and Moderating Role of Hours of Caregiving per Day in Spousal Caregivers* | | |
| --- | --- | --- |
|  | **Stress Over Time** | |
|  | Mean intercept  (Estimate, 95% CI); p-value | Mean slope  (Estimate, 95% CI); p-value |
|  | 18.50 (16.70; 20.29); .008 | 2.69 (2.40; 2.99); <.001 |
| Age | -.10 (-.17; -.03); <.001 |  |
| Sex | 4.74 (3.49; 5.99); <.001 |  |
| Education | .32 (-.20; .84); .228 |  |
|  | **Felt Age as a Predictor of Stress Over Time** | |
|  | Mean intercept  (Estimate, 95% CI); p-value | Mean slope  (Estimate, 95% CI); p-value |
| Felt age | -1.19 (-1.39; -.90); <.001 | -.07 (-.25; .11); .462 |
| Age | -.06 (-.13; .01): .091 |  |
| Sex | 4.24 (3.04; 5.44); <.001 |  |
| Education | .55 (.05; 1.04); <.001 |  |
|  | **Interaction Between Felt Age and Hours of Caregiving Per Day as Predictor of Stress Over Time** | |
|  | Mean intercept  (Estimate, 95% CI); p-value | Mean slope  (Estimate, 95% CI); p-value |
| Felt age | -.75 (-1.13; -.37); <.001 | -.09 (-.46; .29); .651 |
| Hours of caregiving per day | 4.21 (3.06; 5.37); <.001 | .22 (-.97; 1.41); .719 |
| Felt age x hours of caregiving per day | -.21 (-.45; .02); .078 | .04 (-.21; .29); .739 |
| Age | -.05 (-.12; .01); .100 |  |
| Sex | 4.14 (3.02; 5.25); <.001 |  |
| Education | .67 (.21; 1.14); .004 |  |

| **Supplementary Table 9**  *Association of Felt Age and Positive Aspects of Caregiving Over Time, and Moderating Role of Hours of Caregiving per Day in Spousal Caregivers* | | |
| --- | --- | --- |
|  | **Positive Aspects of Caregiving Over Time** | |
|  | Mean intercept  (Estimate, 95% CI); p-value | Mean slope  (Estimate, 95% CI); p-value |
|  | 31.98 (30.64; 33.32); .001 | -.05 (-.30; .19); .659 |
| Age | .09 (-.04; .15); .001 |  |
| Sex | -2.60 (-3.53; -1.67); <.001 |  |
| Education | -1.04 (-1.43; -.65); <.001 |  |
|  | **Felt Age as Predictor of Positive Aspects of Caregiving Over Time** | |
|  | Mean intercept  (Estimate, 95% CI); p-value | Mean slope  (Estimate, 95% CI); p-value |
| Felt age | .18 (.01; .35); .037 | .07 (-.08; .21); .386 |
| Age | .09 (.03; .14); .002 |  |
| Sex | -2.54 (-3.47; -1.61); <.001 |  |
| Education | -1.07 (-1.46; -.69); <.001 |  |
|  | **Interaction Between Felt Age and Hours of Caregiving Per Day as Predictor of Positive Aspects of Caregiving Over Time** | |
|  | Mean intercept  (Estimate, 95% CI); p-value | Mean slope  (Estimate, 95% CI); p-value |
| Felt age | -.09 (-.42; .24); .584 | .05 (-.28; .37); .786 |
| Hours of caregiving per day | -.73 (-1.73; .26); .149 | -.18 (-1.21; .85); .735 |
| Felt age x hours of caregiving per day | .19 (-.01; .40); .064 | -.002 (-.22; .21); .979 |
| Age | .08 (.03; .14); .002 |  |
| Sex | -2.58 (-3.52; -1.64); <.001 |  |
| Education | -1.03 (-1.42; -.64); <.001 |  |

| **Supplementary Table 10**  *Association of Felt Age and Depression Over Time, and Moderating Role of Hours of Caregiving per Day in Adult-Child Caregivers* | | |
| --- | --- | --- |
|  | **Depression Over Time** | |
|  | Mean intercept  (Estimate, 95% CI); p-value | Mean slope  (Estimate, 95% CI); p-value |
|  | 9.03 (2.94; 15.13); .004 | .95 (.22; 1.68); .011 |
| Age | -.21 (-.37; -.05); .012 |  |
| Sex | 2.21 (-1.25; 5.67); .210 |  |
| Education | -.57 (-2.13; .99); .472 |  |
|  | **Felt Age as Predictor of Depression Over Time** | |
|  | Mean intercept  (Estimate, 95% CI); p-value | Mean slope  (Estimate, 95% CI); p-value |
| Felt age | -1.78 (-2.28; -1.27); .316 | -.03 (-.47; .40); .876 |
| Age | -.08 (-.22; .07); .290 |  |
| Sex | 1.83 (-1.18; 4.83); .233 |  |
| Education | .15 (-1.21; 1.51); .829 |  |
|  | **Interaction Between Felt Age and Hours of Caregiving Per Day as Predictor of Depression Over Time** | |
|  | Mean intercept  (Estimate, 95% CI); p-value | Mean slope  (Estimate, 95% CI); p-value |
| Felt age | -.45 (-2.34; -.55); .002 | .15 (-.80; 1.11); .757 |
| Hours of caregiving per day | 2.58 (-.95; 6.11); .153 | 2.40 (-1.82; 6.61); .265 |
| Felt age x hours of caregiving per day | -.44 (-1.17; .30); .243 | -.38 (-1.27; .52); .406 |
| Age | -12 (-.28; .02); .090 |  |
| Sex | 1.49 (-1.63; 4.61); .348 |  |
| Education | -.07 (-1.48; 1.34); .923 |  |

| **Supplementary Table 11**  *Association of Felt Age and Number of Health Conditions Over Time, and Moderating Role of Hours of Caregiving per Day in Adult-Child Caregivers* | | |
| --- | --- | --- |
|  | **Number of Health Conditions Over Time** | |
|  | Mean intercept  (Incidence rate ratio, 95% CI); p-value | Mean slope  (Incidence rate ratio, 95% CI); p-value |
|  | .61 (.21; 1.75); .357 | 1.16 (1.01; 1.34); .039 |
| Age | 1.02 (.99; 1.05); .272 |  |
| Sex | 1.15 (.61; 2.16); .660 |  |
| Education | .88 (.67; .15); .370 |  |
|  | **Felt Age as a Predictor of Number of Health Conditions Over Time** | |
|  | Mean intercept  (Incidence rate ratio, 95% CI); p-value | Mean slope  (Incidence rate ratio, 95% CI); p-value |
| Felt age | .90 (.83; .99); .641 | 1.02 (.93; 1.11); .714 |
| Age | 1.02 (.99; 1.05); .175 |  |
| Sex | 1.11 (.61; 2.03); .737 |  |
| Education | .92 (.70; .20); .542 |  |
|  | **Interaction Between Felt Age and Hours of Caregiving Per Day as Predictor of Number of Health Conditions Over Time** | |
|  | Mean intercept  (Incidence rate ratio, 95% CI); p-value | Mean slope  (Incidence rate ratio, 95% CI); p-value |
| Felt age | .90 (.76; 1.05); .177 | 1.03 (.86; 1.23); .975 |
| Hours of caregiving per day | 1.00 (.57; 1.75); .990 | 1.09 (.51; 2.34); .822 |
| Felt age x hours of caregiving per day | 1.09 (.51; 2.34); .850 | 1.01 (.90; 1.14); .907 |
| Age | 1.02 (.99; 1.05); .163 |  |
| Sex | 1.11 (.60; 2.03); .754 |  |
| Education | .92 (.70; 1.21); .549 |  |

| **Supplementary Table 12**  *Association of Felt Age and Stress Over Time, and Moderating Role of Hours of Caregiving per Day in Adult-Child Caregivers* | | |
| --- | --- | --- |
|  | **Stress Over Time** | |
|  | Mean intercept  (Estimate, 95% CI); p-value | Mean slope  (Estimate, 95% CI); p-value |
|  | 16.29 (10.40; 22.18); <.001 | 1.36 (.54; 2.19); .001 |
| Age | -.07 (-.23; .09); .391 |  |
| Sex | 1.66 (-1.69; 5.02); .331 |  |
| Education | .89 (-.62; 2.41); .249 |  |
|  | **Felt Age as a Predictor of Stress Over Time** | |
|  | Mean intercept  (Estimate, 95% CI); p-value | Mean slope  (Estimate, 95% CI); p-value |
| Felt age | -1.56 (-2.05; -1.07); <.001 | -.07 (-.54; .41); .787 |
| Age | -.01 (-.15; .14); .942 |  |
| Sex | 1.01 (-2.05; 4.06); .519 |  |
| Education | 1.23 (-.14; 2.62); .079 |  |
|  | **Interaction Between Felt Age and Hours of Caregiving Per Day as Predictor of Stress Over Time** | |
|  | Mean intercept  (Estimate, 95% CI); p-value | Mean slope  (Estimate, 95% CI); p-value |
| Felt age | -1.38 (-2.21; -.55); .001 | -.24 (-1.15; .67); .472 |
| Hours of caregiving per day | 4.89 (1.58; 8.20); .004 | -.13 (-4.06; 3.80); .949 |
| Felt age x hours of caregiving per day | -.25 (-.93; .44); .479 | .18 (-.64; 1.01); .665 |
| Age | .01 (-.13; .15); .909 |  |
| Sex | 1.13 (-1.80; 4.06); .451 |  |
| Education | 1.18 (-.14; 2.51); .080 |  |

| **Supplementary Table 13**  *Association of Felt Age and Positive Aspects of Caregiving Over Time, and Moderating Role of Hours of Caregiving per Day in Adult-Child Caregivers* | | |
| --- | --- | --- |
|  | **Positive Aspects of Caregiving Over Time** | |
|  | Mean intercept  (Estimate, 95% CI); p-value | Mean slope  (Estimate, 95% CI); p-value |
|  | 34.50 (30.57; 38.43); <.001 | .16 (-.40; .73); .564 |
| Age | -.14 (-.25; -.03); .010 |  |
| Sex | -2.80 (-5.05; -.55); .015 |  |
| Education | -.80 (-1.81; .73); .125 |  |
|  | **Felt Age as Predictor of Positive Aspects of Caregiving Over Time** | |
|  | Mean intercept  (Estimate, 95% CI); p-value | Mean slope  (Estimate, 95% CI); p-value |
| Felt age | .44 (.08; .81); .016 | -.10 (-.43; .24); .566 |
| Age | -.16 (-.27; -.05); .003 |  |
| Sex | -2.62 (-4.85; -.39); .021 |  |
| Education | -.93 (-1.94; .08); .071 |  |
|  | **Interaction Between Felt Age and Hours of Caregiving Per Day as Predictor of Positive Aspects of Caregiving Over Time** | |
|  | Mean intercept  (Estimate, 95% CI); p-value | Mean slope  (Estimate, 95% CI); p-value |
| Felt age | .60 (-.03; 1.23); .064 | -.22 (-.91; .48); .539 |
| Hours of caregiving per day | .17 (-2.30; 2.65); .890 | -1.10 (-4.08; 1.89); .471 |
| Felt age x hours of caregiving per day | -.11 (-.62; .40); .673 | .11 (-.52; .74); .742 |
| Age | -.17 (-.27; -.06); .002 |  |
| Sex | -2.52 (-4.74; -.30); .026 |  |
| Education | -.85 (-1.86; .15); .096 |  |
